# Randomised and non-randomised studies to estimate the effect of community-level public health interventions: definitions and methodological considerations

**DOI:** 10.1186/s12982-017-0063-5

**Published:** 2017-09-07

**Authors:** Wolf-Peter Schmidt

**Affiliations:** Department of Disease Control, Faculty of Infectious and Tropical Diseases, London School of Hygiene and Tropical Medicine, Keppel St, London, WC1E 7HT UK

**Keywords:** Study design, Cluster randomisation, Public health interventions

## Abstract

**Background:**

The preferred method to evaluate public health interventions delivered at the level of whole communities is the cluster randomised trial (CRT). The practical limitations of CRTs and the need for alternative methods continue to be debated. There is no consensus on how to classify study designs to evaluate interventions, and how different design features are related to the strength of evidence.

**Analysis:**

This article proposes that most study designs for the evaluation of cluster-level interventions fall into four broad categories: the CRT, the non-randomised cluster trial (NCT), the controlled before-and-after study (CBA), and the before-and-after study without control (BA). A CRT needs to fulfil two basic criteria: (1) the intervention is allocated at random; (2) there are sufficient clusters to allow a statistical between-arm comparison. In a NCT, statistical comparison is made across trial arms as in a CRT, but treatment allocation is not random. The defining feature of a CBA is that intervention and control arms are not compared directly, usually because there are insufficient clusters in each arm to allow a statistical comparison. Rather, baseline and follow-up measures of the outcome of interest are compared in the intervention arm, and separately in the control arm. A BA is a CBA without a control group.

**Conclusion:**

Each design may provide useful or misleading evidence. A precise baseline measurement of the outcome of interest is critical for causal inference in all studies except CRTs. Apart from statistical considerations the exploration of pre/post trends in the outcome allows a more transparent discussion of study weaknesses than is possible in non-randomised studies without a baseline measure.

## Background

Public health interventions are often delivered at the level of a cluster, for example a community or larger areas, and not at the individual level. Allocating interventions at cluster-level has methodological, analytical and ethical implications. The development of methods for conducting cluster-randomised trials (CRT) has set a standard in the evaluation of public health interventions [1, 2]. The practical limitations of CRTs and the need for alternative methods continue to be debated [3–8]. The methodology of other study designs is not well defined, and often remains explicitly ignored by textbooks (e.g. [2]). As a consequence, the quality of non-randomised designs may suffer, although they may require an equal or greater amount of thought [8] and methodological rigor [6].

There appears to be no consensus on how to classify study designs evaluating cluster-level interventions. Terms such as before-and-after trial, controlled before-and-after trial and non-randomised trial can be found in reports of public health trials, often without accounting for cluster-level allocation of an intervention. This article reviews how cluster-level allocation affects the classification of study designs and proposes a simple design-based classification of such studies. Key methodological features of different designs are discussed, especially with regard to their impact on the level of evidence potentially obtainable from a study. The article is meant to provide guidance for the planning stage of intervention studies where the intervention is allocated at community level, and is not intended to contribute to the methodology of systematic reviews, which has been reviewed extensively [9–11].

## Analysis

### Study designs for impact evaluations: a brief review of definitions

Habicht and colleagues suggested a framework in which study designs are categorised as adequacy, plausibility and probability evaluations [12]. According to this classification, adequacy studies are basically designs without a control group, plausibility studies are those with a control group not allocated at random, while randomised trials are referred to as probability studies. A more descriptive classification has been published by Kirkwood and colleagues, which distinguishes between pre-post, intervention-control and adopter versus non-adopter comparisons, applicable to cohort, cross sectional and case–control studies [13]. This is a useful approach, since within the different trial designs to evaluate community-level interventions, the traditional definitions of epidemiological studies (case–control, cross sectional, and cohort study) represent tools, not studies in themselves: the outcome in a randomised controlled trial can be assessed using a cross sectional survey or by enrolling a cohort.

The Cochrane Effective Practice and Organisation of Care (EPOC) group proposes four study designs suitable for inclusion into systematic reviews: randomised controlled trials, non-randomised trials, controlled before and after studies and interrupted time series studies (ITS) [9]. Non-randomised trials are defined as trials where the investigator controls allocation, which is not at random. Controlled before-and-after trials are defined by pre- and post-intervention outcome assessment and a non-random group allocation that is not under the control of the investigator. This is in line with a recent UK Medical Research Council definition of natural experiments as a design where allocation is not under the control of the researcher, and where the intervention is not done for the purposes of research [14]. In practice, however, it matters little under whose control allocation was done. It is either random or not. Taking an outcome measure before and after an intervention is also not a suitable defining feature as this is done in many randomised and non-randomised trials.

In this article, it is proposed that most study designs used for the evaluation of cluster-level interventions fall into four broad categories: the cluster-randomised trial, the non-randomised cluster trial (NCT), the controlled before-and-after trial (CBA), and the before-and-after trial without control (BA). Each of these, under certain conditions, has the potential for providing useful or misleading evidence, and the apparent hierarchy in the strength of these designs (CRT > NCT > CBA > BA) needs to be treated with caution [11]. This is in contrast to the Habicht classification, which links these designs to the strength of the obtainable evidence (adequacy, plausibility, probability) [12]. Definitions and key design features of CRTs, NCTs, CBAs and BAs are discussed in the following section, and summarized in Table 1.

**Table 1:** Defining characteristics of cluster randomised trials (CRT), non-randomised cluster trials (NCT), controlled before-and-after study (CBA), and before-and-after trials (BA)

	CRT	NCT	CBA	BA
Defining features	Randomisation of adequate number of clusters to allow statistical between-arm comparison	Non-random allocation of adequate number of clusters to allow statistical between arm comparison	Random or non-random allocation of small number of clusters - too few to allow statistical between-arm comparison	Before and after assessment of outcomes in the absence of a control group
Appropriate setting	Adequate resources, randomisation feasible	Adequate resources, randomisation is politically or logistically not possible	Resource limited evaluations or where number of clusters is naturally constrained (e.g. a district-level intervention in a province that only has 4 districts)	Mass media campaign where no unexposed group can be found
Detectable effect size given adequate sample size	Small	Small to moderate (depending on baseline comparability and temporal stability of outcome in control arm)	Moderate to large (depending on baseline comparability and temporal stability of outcome in control arm)	Large
Number of clusters	At least 4–6 clusters per arm, higher if effect size is small	At least 4–6 clusters per arm, higher if effect size is small	At least 2 clusters per arm, unless outcome is assessed repeatedly before and after	Study power is determined by number of participants and number of pre/post measures
Baseline measure of outcome of interest	Not required, but may increase study power and allow adjusting for imbalances	Required	Required	Required
Outcome assessment at multiple time-points	Not required, but may increase study power	Usually not required, but may increase study power	Desirable, required if there is only one cluster per arm	Required
Statistical analysis	Direct comparison between intervention and control	Direct comparison between intervention and control, by adjusting for baseline measure of outcome, by calculating change scores or by calculating the difference-in- difference	Comparison before versus after; control arm only serves to demonstrate absence of trends. Analysis of slope and intercept of trends in the outcome measure (if multiple pre/post measures are available)	Comparison before versus after. Analysis of slope and intercept of trends in the outcome measure (if multiple pre/post measures are available)
Special types	Stepped wedge design Trials using systematic allocation (“quasi-randomised”)	Controlled interrupted time series analysis Per-protocol analysis within a CRT		Interrupted time series analysis Pre/post adopter versus non-adopter analysis

### Trial designs: key methodological features

#### Cluster randomised trial

A CRT as defined here needs to fulfil two basic criteria: (1) the intervention is allocated at random or using a quasi- random method of systematic allocation, and (2) there are sufficient clusters to allow a statistically meaningful comparison between intervention and control. If fewer than 4 clusters are allocated to the intervention and control each (5–6 in a pair-matched trial), then the statistical between-arm comparison is not informative as there is no chance for the *p* value to be low, e.g. lower than 0.05 [1, 13]. A trial in which too few clusters are allocated to allow statistical between-arm comparison is not a CRT by this definition (in contrast to the EPOC definition [9]).

The methodology of CRTs has been described in depth [1, 2, 15]. The key difference between individually randomised and cluster-randomised studies lies in the loss in study power due to cluster-randomisation, often expressed as the design effect [1, 2, 15]. The design effect is the factor by which the sample size needs to be multiplied to account for clustering. The design effect can be unpredictable for outcomes with a high spatial or temporal variability, as in the case of many infections [16]. Underestimating the design effect occurs even in otherwise well-planned studies. For example, in the large-scale ZAMSTAR trial testing two tuberculosis case-finding interventions, the design effect was considerably higher than expected [17]. The effect sizes found were of public health relevance, but owing to the underestimation of the design effect the width of the confidence intervals precluded meaningful interpretation, at a cost of US$15m. Unless reliable design effect estimates are available or the effect size of interest is large, CRTs can be an expensive gamble. However, underpowered CRTs have one important advantage over underpowered NCTs, CBAs or BAs: effect estimates in well-designed and well conducted CRTs can be regarded as unbiased even if the confidence intervals are wide. Results from several inconclusive CRTs can be pooled in meta-analysis to improve precision. Statistically, meta-analysis is much more straightforward for CRTs than for non-randomised studies where pooled effects reflect the average amount of confounding, whereas in CRTs confounding can only be due to chance. Unlike in CRTs, a large number of studies included in a meta-analysis of non-randomised studies does not minimise confounding.

While all study designs provide more convincing evidence if the effect size of interest is large, the CRT remains the only study design suitable to investigate small effects. Both the confidence interval of the effect size and the risk of confounding can be minimised by increasing the sample size (in particular the number of clusters), sometimes allowing the detection of very small effects [18]. All other study designs are at higher risk of confounding, the size of which is independent of the sample size. Even if confounding can be minimised by statistical methods, the potential for residual confounding is likely to be larger than an expected small intervention effect.

As will be discussed in more detail below, the CRT is the only study design that does not require a baseline measure of the outcome to minimise confounding, although a baseline can help to improve study power, explore eventual imbalances and adjust for these if appropriate. Imbalances between study arms can be assumed to have arisen by chance, unless proven otherwise. Baseline measurements are costly, may cause reactivity in the study population due to repeated surveying and may already be outdated by the time a delayed intervention is delivered. If no baseline survey is needed, the investigators are in the comfortable position of letting the implementers work according to their schedule, and use the time to develop study procedures in a subset of the trial population or in external pilot clusters.

A disadvantage of CRTs is that for ethical reasons, participants often (but not always [19]) may need to be told that they are part of a trial, possibly altering their behaviour and response to questions [20–22]. This may be a considerable problem especially in trials that are neither blinded nor use an objective outcome measure. Several meta-analyses have shown that such trials produce estimates that are severely affected by responder and observer bias [20, 21]. These trials are the most problematic for the public since randomised trials carry a large weight in decision making, while it is the process of informed consent (usually required in a randomised trial) that may contribute to bias [21]. Not all may be lost for unblinded trials with a subjective outcome in situations where the purpose of the outcome assessment can be hidden from the study participants, for example by presenting it as a general health survey. In this context, the unit of treatment allocation may be important. If an unblinded intervention evaluated using a subjective outcome (e.g. self-reported symptoms) is allocated to small clusters (e.g. households), the link between the intervention and outcome assessment may be obvious to participants and their responses are likely to be biased. If allocation is done at community level (e.g. a large scale government-led public health intervention), followed by surveys to assess disease symptoms, then the link between intervention and outcome assessment may not be obvious. For example, it has been demonstrated through systematic reviews that unblinded trials of interventions of point-of-use (household) water treatment with reported diarrhoea symptoms as outcome are severely biased, suggesting a 50% reduction in diarrhoea (despite poor compliance), while blinded trials showed no reduction [21]. In contrast, several CRTs on community-level sanitation (an unblinded intervention) with the same outcome (self-reported diarrhoea symptoms) and equally poor compliance with the intervention showed no effect at all [23–25]. In both the point-of-use water treatment and the sanitation trials, compliance with the intervention was very poor. For both interventions, a true effect would have been biologically implausible. The absence of an observed effect in the sanitation trials may therefore be regarded not only as evidence for absence of a true effect but (in contrast to the water treatment trials) also as evidence for lack of responder bias, possibly because participants did not link the health surveys to the intervention or did not expect any benefits from giving false information [23–25].

#### Non-randomised cluster trial

In a NCT, statistical comparison is made across trial arms as in a CRT, but treatment allocation is not random. Allocation is done by the investigator or the implementer, e.g. based on logistics or needs. The EPOC definition of non-randomised trials requires that the investigator controls allocation [9]. In the definition used here, allocation is not random and it does not matter who allocates. An implementer may decide to deliver an intervention in ten villages, and an evaluator may choose ten suitable control villages for comparison [26, 27]. With notable exceptions [28], participants may not need to be explicitly told that they are part of a trial. Trial procedures may more easily be camouflaged as general demographic and health surveys than in a CRT, which may reduce responder bias.

NCTs need to demonstrate that intervention and control arms are comparable. Unlike in CRTs, imbalances are not due to chance until proven otherwise (which is usually impossible). Most often, baseline characteristics are used to adjust for imbalances. Baseline variables may include (1) demographic and socio-economic characteristics and other covariates potentially associated with outcome and intervention, and (2) a baseline measure of the outcome of interest. These two measures need to be clearly distinguished as it can be argued that adjusting for the latter is likely to be more effective than for the former. In a sense, baseline variables are a predictor of the baseline measure of the study outcome, which in turn is a predictor of the outcome at follow-up. Then, conceptually, the outcome in the control group is used as a proxy to estimate the outcome in the intervention group had it not received the intervention (the “potential outcome” [29]). Hence, the baseline measure of the study outcome can be regarded as more proximate to the potential outcome than other baseline variables. Investigating trends of the study outcome from baseline to follow-up is a fairly transparent way of exploring whether baseline imbalances may have affected the effect estimate, as the trends in the outcome in different study arms can be openly discussed. If there is no baseline measure of the study outcome, then one can only compare other baseline variables (e.g. socio-demographic characteristics) between intervention and control, and then use these variables in a multivariable statistical model. Such models, however, usually represent a black box with an unknown amount of residual confounding [30]. It can be argued that the only way to make a NCT convincing is to obtain a precise baseline measurement of the study outcome and use it in the final analysis [31]. No amount of multivariable adjustment or matching of other variables, even if done with great care [27, 32], can replace the value of a precise baseline measure of the study outcome.

Statistical methods to account for baseline measure are imperfect and continue to be debated [33, 34]. Methods include the analysis of covariance (or lagged regression) method [33, 34], the analysis of change scores [4, 33, 34], and the exploration of the interaction between treatment allocation and time point [35]. In the analysis of covariance method, regression models are used that include the baseline measure as just another explanatory variable. The analysis of change-scores is based on between-arm comparison of the difference between the outcome at follow-up and the outcome at baseline, measured in the same individual, or, in cluster-level analysis [36], in the same cluster [34, 37]. The interaction approach is required if different individuals are measured at baseline and follow-up, and is calculated as the interaction term between treatment allocation and time-point (e.g. baseline vs. follow-up) [38]. The effect estimates produced by the change score and the interaction approaches are sometimes referred to as Difference-in-Difference (DID) [35]. All three methods work well if baseline imbalances are relatively small, but become problematic if imbalances are large [4, 34, 37], which is fair enough as in this case trial arms are probably not comparable to start with. The regression approach works well if baseline and follow up measures are highly correlated, which is often the case for continuous variables such as child anthropometrics or blood pressure. The regression approach is problematic for binary outcomes. Binary outcomes measured at two different time points (e.g. diarrhoea prevalence or breastfeeding in the past 24 h) are rarely highly correlated. Adjusting for a baseline measure showing only a low or moderate correlation with the follow up measure leads to regression dilution bias, and failure of the regression model to adequately adjust for any baseline imbalance [4]. The change score approach may be preferable in this situation [33]. It is important to maximise between-arm comparability and not solely rely on statistical methods to achieve balance, since the three methods mentioned above each rely on a number of assumptions. Choosing comparable control clusters is central in the design of NCTs (and similarly CBAs), for example by matching. Various matching methods can be applied to achieve comparability [31], including using publicly available census data [27, 32]. The most promising approach may be to match intervention and clusters according to the baseline measure of the outcome of interest, which however may not yet be available at the time of recruitment.

A special case of NCT is the controlled interrupted time series study (CITS), which can provide high quality evidence [39–41]. Across a number of intervention and control clusters many repeated measurements of the outcome of interest are taken before and after the intervention. Usually, CITS require the use of regularly collected routine data, which often are only available at the level of large administrative units (e.g. states, provinces). The analysis focuses on whether a certain change in the outcome has taken place after the intervention in the intervention but not the control clusters. To include intervention and control clusters in the same model, they need to be reasonably comparable. CITS have the advantage that the requirement of including at least 4–6 clusters per arm [1, 13] may be relaxed by including a fixed effect for cluster intercepts to control for time-invariant differences between clusters. It may not be necessary to consider random variation in the intervention effect across clusters.

#### Controlled before-and-after study

The key feature of a CBA as defined here is that intervention and control arms are not compared statistically. Rather, baseline and follow-up measures of the outcome of interest are compared in the intervention arm, and separately in the control arm. The control arm only serves to get an idea of what the trend in the intervention arm might have been in the absence of an intervention. Whether or not the intervention is allocated at random is of no relevance for this definition. For example, a trial allocating a total of four villages at random to intervention and control (2/2) can only be analysed pre/post in each arm, because between-arm comparison is statistically uninformative, as there is no chance of observing low p-values [1, 13]. This definition is in contrast with the EPOC definition which defines a CBA as a trial where before and after measures are taken and where allocation is non-random and outside the control of the investigator [9], equivalent to the MRC definition of a natural experiment [14].

Design and interpretation of CBA studies have been described [5], often disregarding the issue of cluster-level allocation. In CBAs, the study outcomes can be compared statistically between different points in time before and after the intervention, but only separately for intervention control clusters, not between them. The comparison between intervention and control arm can only be done informally without statistical methods, e.g. by producing a graph. Not being able to calculate a confidence interval or p-value for between-arm comparison is unsatisfying, and usually excludes such studies from formal meta-analyses. In some small CRTs or NCTs with for example 4 or 5 clusters per arm, statistical between-arm comparison is theoretically possible but may have low power. In one trial in India with just 5 clusters per arm, the investigators chose to analyse the data as a CBA, with the direct comparison serving only as a secondary analysis to enable future meta-analyses [42]. The advantages of this approach are unclear and require further study.

**Fig. 1:**
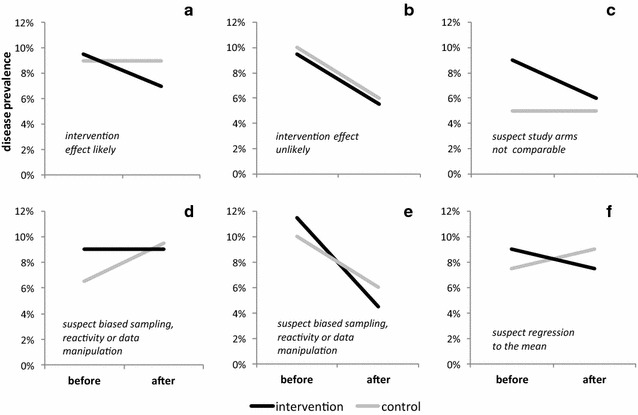
Trend interpretation in non-randomised cluster trails (NCT) and before-and-after trails with control group (CBA): **a** good balance, no trend in control arm; **b** good balance, strong trend in control arm; **c** poor balance, no trend in control arm; **d** poor balance, strong trend in control arm; **e** poor balance, erratic trends; **f** poor balance, opposing trends

Trend interpretation is critical in CBAs. Similar considerations apply to NCTs, but in CBAs one cannot even use statistical analysis to compare trends across arms. An often cited requirement in NCTs and CBAs is the parallel-trend assumption, assuming that intervention and control arms would have shown the same trend from baseline to follow-up in the absence of an intervention. However, especially small scale CBAs can often only be meaningfully interpreted if no trend at all is observed in the control arm, i.e. if the outcome does not change over time in the absence of an intervention. A moderate or even large change in the control arm from baseline to follow-up may more often indicate a methodological problem in the study procedures than a true change. The absence of a trend in the control arm makes a NCT but especially a CBA much more credible. Some possible trends observable in CBAs or NCTs are shown in Fig. 1. All scenarios share a similar Difference-in-Difference of about 3% (except B), but are not equally convincing [5]. Scenario A most strongly suggest an intervention effect, as in the control prevalence is similar at baseline and remains constant at follow-up. In scenario B, intervention and control start at similar prevalence values that decrease in parallel, suggesting absence of an independent intervention effect. This scenario is often encountered in situations of rapid economic development, the health benefits of which overshadow public health interventions [43, 44]. In scenario C, prevalence is very different at baseline, suggesting that the two arms are not comparable. Caution is warranted in interpreting the DID estimate as an intervention effect. This applies even more to Scenario D where there is no change in the intervention arm and a prevalence increase in the control arm. Lack of baseline comparability or poor data quality may well be the cause for the observed trends. In CBAs and NCTs, considerable skill (and sometimes a bit of luck) is required to identify control clusters with comparable outcome levels at baseline.

Strong and erratic temporal trends in the outcome measure can be natural (e.g. in infectious diseases), or may indicate a change in the sampling approach or survey tools from baseline to follow-up, or data manipulation (Scenarios E and F). In NCTs and CBAs, the evaluation of trends is greatly compromised by pre/post changes in the data collection methods, highlighting that great care must be taken to apply the exact same sampling procedures and outcome assessment tools at all time points.

A trend in the opposite direction (Scenario F) raises the possibility of regression to the mean [45]: the intervention clusters may have been chosen because prevalence was temporarily high prior to the intervention, indicating a need for an intervention. The control area may have been excluded from the intervention because of temporarily favourable indicators. Absence of regression to the mean effects is best demonstrated by including measurements at different time points both before and after the intervention.

These examples demonstrate the many obstacles faced by trend analyses in the context of NCTs and especially CBAs to identify true intervention effects—the price of non-random allocation. They highlight the importance of rigorous study procedures to ensure comparability of baseline and follow-up surveys. They also show the value of comparing trends between trial arms, which allows a fairly transparent discussion about the merits and limitations of a particular study. Consider a NCT, where no baseline data are available in scenarios A to F, and multivariable regression analysis is used to address confounding. Even if as recommended [4] investigators carefully adjusted for confounders, reported their methods thoroughly and were conscious and critical of the assumptions they made, the analysis would still be a black box. Neither those who argue in favour of a true effect, nor those arguing that all is due to confounding have much in their hands to support their views.

#### Before-and-after study

This is a CBA without a control group. One or several measures of the outcome of interest are taken at baseline and follow-up, and compared. The absence of a control arm makes it difficult to support the assumption of an absence of a strong secular trend. In this definition (and in contrast to EPOC [9]), ITS studies are a special case of a BA. A typical scenario for a BA is the evaluation of a mass media campaign that targets a whole population, leaving no one to serve as control.

Temporal variability is even more problematic in BAs than they are in CBAs and NCTs, since in the absence of a control arm we do not know how variable the outcome would have been without an intervention. It may therefore not be possible to judge how “bad” the study was. However, some outcomes are naturally stable and are potentially suitable for BAs, but even here confounding is possible. Consider for example the mass media adverts to increase handwashing during the H1N1 pandemic in 2009 [46], where it was hard to distinguish between campaign effects, and the effect of the general anxiety altering people’s behaviour.

One method to increase the validity of a BA is to take several measures of the outcome of interest at baseline and follow-up, ideally to demonstrate a reasonable stability of the outcome of interest pre-intervention and, if the intervention is successful, at a different level post-intervention. If many before and after measurements are available, ITS may allow obtaining statistically robust estimates [47, 48]. In their simplest form, ITS studies assume a common slope over time before and after an intervention and explore changes in the intercept at follow-up. Uncontrolled ITS analysis addresses temporal variability but not the risk of confounding as in the H1N1 example (unlike CITS).

A further way to improve BAs lies in combining it with an adopter versus non-adopter comparison. Adopters are usually defined as those complying with the intervention or at least having been exposed to the intervention. Post-intervention adopter versus non-adopter comparisons are often used to evaluate mass media campaigns e.g. comparing outcomes in those that have seen a TV ad with those that have not seen it [13, 49]. Post-intervention adopter versus non-adopter comparisons carry a high risk of confounding, as it is not clear how similar outcomes in adopters and non-adopters would have been without an intervention. If, however, the same individuals are surveyed before and after the intervention, then trends in the outcome can be assessed separately for adopters and non-adopters, allowing an estimate of the risk of confounding. This design then resembles a CBA study, but is methodologically different as there is no pre-planned or otherwise well-defined group of control clusters. Therefore it is defined here as a subtype of BA, not CBA.

### Study designs and the potential for obtaining relevant evidence

Evaluations of public health interventions at community level need to fulfil at least one of the following three criteria: (1) randomisation of a sufficiently large number of clusters to allow statistical between-arm comparison (RCT), (2) if this is not possible, a precise baseline measure of the outcome of interest to assess baseline comparability and to study trends from baseline to follow-up in the absence of an intervention (NCT, CBA), (3) if there is no control group, multiple measures of the outcome of interest at baseline and after the intervention (BA).

Compared to CRTs, NCTs, and in particular CBAs and BAs are problematic study designs, and likely to be most convincing if (1) the effect size is large; (2) the before-after trends are consistent across the majority of intervention clusters; (3) the outcome at baseline is approximately similar between intervention and control (CRT, NCT, CBA); 4) there is no major trend in either direction observable in the control clusters (NCT, CBA).

Different trial designs may be applicable at different stages of an evaluation: for example, a campaign to promote exclusive breastfeeding (EBF) may need to achieve an exposure of say 90% of the target population to increase motivation to practice EBF from say 20 to 70% (a difference of 50 percentage points) to achieve an actual increase in EBF from say 20 to 50% (a difference of 30 percentage points), to result in a reduction of diarrhoea prevalence from 10 to 8% (a difference of two percentage points). With each step, the effect size of public health relevance decreases: a reduction in diarrhoea of 2 percentage points would be an important public health goal, while we would not be content with a 2 percentage points difference in intervention coverage or EBF. Similar arguments can be made for the evaluation of biomedical interventions such as micronutrient supplements, where high compliance is needed to achieve a large change in serum-micronutrient levels, which may lead to a moderate change in subclinical disease and a small change in morbidity or mortality [50]. The smaller the effect size of interest, the larger the sample size and the better the study quality needs to be. In addition, the scope for confounding is likely to increase the more downstream an outcome is situated, as the number of potentially uncontrolled causal influences on the endpoint tends to add up.

## Conclusion

Some study designs are likely to confuse public health decision making, rather than inform it [51]. These rely on multivariable analysis to control for confounding and include: NCT without a baseline measure (e.g. conducted as cross sectional surveys or cohort studies post-intervention comparing intervention and control areas); cross sectional surveys post-intervention comparing adopters and non-adopters e.g. to study the effect of a mass media intervention; BAs with only one measure of the outcome before and after the intervention. Case control studies are usually unsuitable for the evaluation of community level intervention because of a high risk of confounding and bias, and the difficulty in dealing with the community level exposure allocation [13]. Table 2 outlines a categorisation of study designs and the expected quality of evidence as a rough guide to help in the planning of an evaluation. There are situations where a study is little more than occupational therapy for field researchers. While there are many design features that threaten the quality of randomised trials, a landmark meta-analysis of clinical trials by Savovic and colleagues has demonstrated that in particular the combination of lack of blinding and the use of a subjective outcome strongly impairs validity [20], and this may particularly be the case for public health interventions [21]. CRTs are the preferable study design provided that either the outcome is objective or allocation is blinded to participants and assessors. If this is not the case, then a CRT may not be the “gold standard” and should not carry more weight in health policy than NCTs, CBAs or BAs. It may often be better not to do such a trial, unless the purpose of the trial can be successfully hidden from study participants. Further, a precise baseline measurement of the outcome of interest is critical for causal inference in all studies except CRTs, to the point where non-randomised studies without a baseline measure may not be worth doing (Table 2).

**Table 2:** Study design and the potential level of evidence

Study type	Designs with the potential to provide strong evidence	Designs with the potential to provide reasonable evidence	Designs likely to provide weak or misleading evidence
CRT	CRT that is blinded to participants and investigators OR uses an objective outcome measure	CRT that is not blinded to the participants AND uses a subjective outcome measure AND study participation is not obvious to participants (see text)	CRT that is not blinded to the participants AND uses a subjective outcome measure AND study participation is obvious to participants (see text)
NCT	NCT with very good balance of the outcome of interest at baseline across arms NCT conducted as controlled interrupted time series analysis	NCT with imbalance of the outcome of interest at baseline across arms AND no major trend in the control arm	NCT with imbalance of the outcome of interest at baseline across arms AND a major trend in the control arm NCT without baseline measure of the outcome of interest
CBA		CBA with reasonable balance of the outcome of interest at baseline across arms AND no major trend in the control arm CBA with only one cluster per arm AND multiple outcome measures before and after the intervention	CBA with imbalance of the outcome of interest at baseline across arms OR major trend in the control arm CBA with only one cluster per arm AND single outcome measure before and after the intervention
BA		BA with multiple outcome measures before and after the intervention BA with adopter/non-adopter comparison before and after the intervention BA conducted as interrupted time series analysis	BA with a single outcome measure before and after the intervention BA using post-intervention adopter/non-adopter comparison without baseline

The main argument in favour of this view is not purely statistical. Rather, the exploration of pre/post trends in the outcome allows a more transparent discussion of study weaknesses than it is possible in studies relying on adjustment for other, more distal confounders.

## Abbreviations

BA: before-and-after study
CBA: controlled before-and-after study
CITS: controlled interrupted time series
CRT: cluster-randomised trial
DID: difference-in-difference
ITS: interrupted time series
NCT: non-randomised cluster trial
